# Remote post-mortem radiology reporting in disaster victim identification: experience gained in the 2017 Grenfell Tower disaster

**DOI:** 10.1007/s00414-019-02109-x

**Published:** 2019-06-28

**Authors:** Guy N. Rutty, Mike J. P. Biggs, Alison Brough, Bruno Morgan, Philip Webster, Ann Heathcote, Jessica Dolan, Claire Robinson

**Affiliations:** 1 East Midlands Forensic Pathology Unit, University of Leicester, Robert Kilpatrick Building, Leicester, LE2 7LX UK; 2 Cellmark Forensic Services, Buckshaw Link, Ordnance Rd, Buckshaw Village, Chorley, PR7 7EL UK; 3 University of Leicester Imaging Department, University Hospitals of Leicester, Leicester Royal Infirmary, Leicester, UK; 4 Alliance Medical Ltd, ICENI Centre, Warwick Technology Park, Warwick, CV34 6DA UK; 5 Imaging Department, University Hospitals of Leicester NHS Trust, Leicester, UK

**Keywords:** Forensic, Grenfell Tower, Mass fatality, Disaster victim identification, Post-mortem computed tomography, Remote radiology reporting, INTERPOL

## Abstract

On 14 June 2017 at 00:54 h, the worst residential fire since the conclusion of the Second World War broke out in Flat 16, 4th floor of the 24-storey residential Grenfell Tower Block of flats, North Kensington, West London, UK. Seventy-one adults and children died, including one stillbirth. All victims of the Grenfell Tower disaster who died at the scene underwent post-mortem computed tomography (PMCT) imaging using a mortuary-sited mobile computed tomography scanner. For the first time, to the authors’ knowledge, the disaster victim identification (DVI) radiology reporting was undertaken remote to the mortuary scanning. Over an 11-week period, 119 scans were undertaken on 16 days, with up to 18 scans a day. These were delivered to a remote reporting centre at Leicester on 13 days with between 2 and 20 scans arriving each day. Using a disaster-specific process pathway, a team of 4 reporters, with 3 support staff members, trialled a prototype INTERPOL DVI radiology reporting form and produced full radiology reports and supporting image datasets such that they were able to provide 96% of prototype DVI forms, 99% of image datasets and 86% of preliminary reports to the DVI teams in London within one working day of image receipt. This paper describes the first use of remote radiology reporting for DVI and exemplifies how remote PMCT reporting can be used to support a DVI process of this scale.

**Electronic supplementary material:**

The online version of this article (10.1007/s00414-019-02109-x) contains supplementary material, which is available to authorized users.

## Introduction

Since Rutty et al. [1, 2] first reported the use of mobile post-mortem computed tomography (PMCT) in a multiple fatality incident in 2007, PMCT has slowly become more widely utilised in disaster victim identification (DVI), as it becomes more globally integrated into autopsy practice. The role of PMCT in DVI is exemplified by the paper of O’Donnell et al. [3] concerning the 2009 Victorian bushfires and more recently in its role in the MH17 investigation [4, 5]. The International Society for Forensic Radiology and Imaging (ISFRI) DVI working group has issued international guidance on the use of radiology, and specifically PMCT, in DVI through a series of ISFRI positional statements [6–9].

The concept of remote reporting of radiology in DVI was first suggested by Rutty et al. [10] in 2009. To date, certainly within the UK, outside Exercise Hounslow, the 2011 multinational mobile PMCT DVI reporting exercise, remote reporting has not, to the authors’ knowledge, been utilised in a live DVI incident. Remote telebiopsy for use in contaminated mass fatalities was suggested by Ebert et al. [11] although again, to the authors’ knowledge, this has not been used in a live DVI incident. Finally, although INTERPOL promotes the use of radiology within the DVI process, there had not been a specific INTERPOL post-mortem Pink form to record the findings of any radiology examination undertaken during the DVI process.

All victims of the Grenfell Tower disaster, London 2017, who died at the scene, underwent PMCT using a mortuary-sited mobile computed tomography (CT) scanner. For the first time, to the authors’ knowledge, the DVI radiology was reported remotely. We describe how this was achieved including the data exchange to the reporting team and the information that was provided back to the mortuary to assist the DVI process. We illustrate the process pathway that was developed at Leicester, explaining the medico-legal importance of such a process pathway, and describe the reporting forms that were utilised including the development and testing of the new INTERPOL radiology reporting form so that others can learn from our experience.

## Materials and method

On 14 June 2017 at 00:54 h, the worst residential fire in the United Kingdom (UK) since the conclusion of the Second World War started in Flat 16, 4th floor of the 24-storey residential Grenfell Tower Block of flats, North Kensington, West London, UK. Of those residents at the time within the 129 flats, sixty-nine adults and children died in the tower with 2 further deaths in the hospital, one child and one stillbirth. The stillbirth underwent a consented post-mortem examination, as the death did not come under coronial jurisdiction.

### Mobile computed tomography scanner deployment

As part of the UK-DVI deployment, an Alliance Medical mobile CT scanner (https://www.alliancemedical.co.uk/ last visited November 2018) was deployed to the Westminster public mortuary on 15 June 2017 where the first fatalities were scanned that day. To ensure evidential integrity, the body bags were not opened within the scanner. As in the Victorian bushfire incident, all presumed human remains were scanned, no matter how small or admixed with fire debris. Initially the scanner was on-site for 7 days. As the recovery slowed, scanning was provided as required to ensure cases had been scanned and reported ahead of examination in the mortuary.

### CT scanning protocol

Scanning was completed using a GE Optima CT660 scanner. A pre-agreed scan protocol developed from previous PMCT DVI experience was used and programmed in to the scanner (120 kV, 120–350 mA, largest field of view). Images were reconstructed at 0.625 mm on 0.625 mm for the head and neck and 2.5 mm on 1.25 mm for the chest, abdomen, pelvis and leg scans, all on a bone and soft tissue algorithm.

A process was established to ensure all scans were completed to the same high standard and that the requirements of all professions working in the mortuary were satisfied. The deceased and their paperwork (the INTERPOL ‘Pink book’) were brought to the scanner by dedicated body movement police officers. Each scan was completed using the INTERPOL number assigned at the time of recovery as the identifier. All bags were scanned in their entirety to ensure any loose fragments in the bags were scanned. If the deceased were largely intact, the scans were separated into 3 segments as the scan length was limited to 1400 mm. The head and neck, chest, abdomen and pelvis (including as much of the arms as possible) and legs (from above the acetabulum to toes) were scanned in each case. If there was disruption to the body, or it was unclear what the contents were after the initial scout views, the bags were scanned in two blocks, rotating the bag between the scans. A landmark was identified in each case to ensure the two scan blocks overlapped.

The body movement officers completed the Pink books while the radiographers were scanning. The radiographers signed the paperwork, and two DVDs of the DICOM data were produced for police evidence once the scan had been completed. The same prototype ‘INTERPOL’ DVI form as used by the remote radiology reporting team was trialled by the radiographers. Initial comments were made on it as to whether the remains may be paediatric or juvenile, the presence of any dentition, any identifying features (including wallets, mobile phones or jewellery) and anything that could pose a risk to the mortuary team. The form was returned to the mortuary with the deceased. This was provided for guidance only with the caveat that a DVI radiology and full radiological report would be provided later by the remote reporting team.

On two occasions, forensic odontologists and anthropologists requested further scanning to assist with their examinations. Due to the collapse of parts of the building, foreign objects were frequently seen in the bags. These repeat examinations were completed after these items had been removed in the mortuary which made locating bone and dentition easier. These scans were particularly useful if items that needed to be recovered from disrupted bodies had moved in the bags during body movement procedures.

### Data transfer

At the time the CT scanner was deployed, rather than send a reporting team to the mortuary, it was decided to activate a remote radiology reporting team at the East Midlands Forensic Pathology Unit (EMFPU), University of Leicester. This minimised personnel on-site as the space in the mortuary was limited and enabled the team to continue supporting both forensic pathology and clinical post-mortem radiology services locally.

Thus, DICOM image data from the CT scanner needed to be transferred to Leicester. The scans completed on day one were burnt onto DVDs which were then hand delivered to Leicester by 19:43 h. All DVI forms were completed by 21:14 h with the full radiology reports completed by 23:15 h the same day. All reports and images were sent to London by the support staff the following day.

Due to the data size and the impracticalities of delivering the data in this way, a means of transferring the data digitally had to be established. Securing sufficient and consistently reliable uploading speed to transfer the scans in a timely manner proved challenging even with the assistance of a specialist unit from an international communications company. The eventual solution involved using elements of the Internet, not readily available without specialised help from the British Telecommunications PLC (BT) Emergency Response Team, dedicated purely to uploading scans. Subsequent data was then sent to Leicester using a secure file drop system. The report and electronic reconstructed case images were transferred back to London through a secure email system. Hard copy backup DVDs of all cases were securely delivered to Leicester. To ensure that colour versions of the reconstructed images were available to the mortuary teams, hard copy colour backups of the reconstructed case images were printed at Leicester and transferred to London in batches. Further reconstructed case images were also produced when requested by the different specialists in the mortuary to assist with their investigation.

### Process pathway

To ensure consistency in the reporting process, image capture, data storage and report output transfer to London, a reporting process pathway was rapidly developed and agreed by the reporting team. A copy of the process pathway is provided with the online resource. By following this agreed process, the reporting team and support staff ensured that no critical stage of the pathway was omitted. It also provided a documented continuity of evidence pathway for any future criminal, civil or coroner’s court procedures.

### Reporting team

The reporting team comprised two forensic pathologists, a forensic radiologist and a forensic anthropologist, all of whom had a minimum of 6-year experience of using PMCT in their clinical practice, research and teaching. The forensic pathologists and anthropologist had both national and international DVI experience. A paediatric radiologist was not utilised on this occasion as issues relating to the identification of children were dealt with by the presence of an anthropologist experienced in CT scanning of the developing skeleton.

Prior to this incident, an ‘INTERPOL’ radiology reporting form did not formally exist. A form was in development, and so the prototype was trialled for each case by the radiographers in London and both pathologists in Leicester. The reporting of the pathological findings followed the normal forensic PMCT practice within the EMFPU for DVI incidents. A forensic pathologist with the assistance of the anthropologist completed the prototype DVI form and then produced a handwritten primary radiology report and image set which, following typing, then went to the radiologist for confirmation of findings and further comment. A final report agreed between the radiologist and case pathologist was then produced in each case. This was achieved using either the routine EMFPU PMCT reporting form or a new so-called ‘remains form’ depending on the nature of the case [12].

The reporting team was supported by three members of the EMFPU support staff. These staff transcribed all handwritten to typed reports on the same day of production, coordinated the production of the final agreed reports and prepared, logged and sent all data packages to London prior to archiving copies of all the materials locally. The normal day-to-day autopsy work of the EMFPU was undertaken by the other forensic pathology and support staff members of the unit and was managed so that it was not affected by the work of the radiology reporting team.

### Remote reporting

The EMFPU has a dedicated PMCT teaching and training room that is used for teaching the post-graduate courses in forensic and natural death radiology. This room was used for the primary reporting of the Grenfell Tower cases. Each case on receipt at the EMFPU was stored onto an Apple Mac computer, which acted as a local data store. From this, the two pathologists and the anthropologist were able to access either the same case simultaneously or separate cases. Reporting was undertaken upon Mac computers using OsiriX MD v.7.02. Image interpretation was assisted by post-processing of image sets to create multiplanar reformatted (MPR) images, three-dimensional (3D) surface shaded display (SSD) volume-rendered reformats for bones and 3D maximum intensity projection (MIP) algorithm with colour coding for high-density picture elements (pixels). Assessment for metallic objects was undertaken using the surface rendering function set to metal default to help flag up metallic items. A curved multiplanar reformat simulating the standard dental panoramic radiography (orthopantomography (OPT)) was produced in cases where the jaws could be identified to facilitate odontological assessment.

### Documentation of findings

For each case, where possible, the reporting team provided, through the prototype ‘INTERPOL’ and pathology reporting forms, information regarding the identification of the deceased including estimation of the age, sex, height and weight of the deceased along with the presence, absence and location of clothing and personal possessions. Piercings sites and hairstyles, where observed, were recorded. The presence of jaws, individual teeth and dental reparative work was recorded. Comment was made on the presence and correct placement of any medical interventional devices that had been placed within or onto the deceased during resuscitation attempts as well as the presence of any natural disease, fire and non-fire trauma. The presence of hazards which could injure a DVI team member was also recorded. A summary was provided of the findings to assist the DVI team along with an abbreviated set of reconstructed key images, which followed the suggested minimal dataset for biological profiling of Brough et al. [13] along with images of any potential identifying features such as personal possessions, medical prosthesis or pre-existing natural disease. On this occasion, as it was known that all cases were to undergo a police DVI, pathological, anthropological and odontology examination at the mortuary, a cause of death was not provided by the reporting team.

## Results

### Time scale

Over an 11-week period, 119 scans were undertaken on 16 days, with up to 18 scans a day. These were delivered to Leicester over 13 days, with 2 to 20 scans arriving on each day.

The nature of scanning changed as the time post-incident progressed and reflected the order of body recovery. On day one, seven scans were completed, with the principal findings being non-fire-related trauma. On day two, the first cases of heat-related trauma were seen. By scan day three, the observed severity of heat-related trauma had increased, and partial remains were imaged. Day four saw remains requiring anthropological assessment and beyond day seven remains were increasingly fragmented. Two cases were scanned twice. Three scanned cases were identified to be purely non-human debris. In three cases, the fire-related ‘pugilistic’ position of the deceased meant part of the upper or lower limb could not be scanned because they were outside the field of scanning, despite attempts to reposition the bag.

### Reporting time scales

The prototype ‘INTERPOL’ DVI and primary pathology report was produced for 77 cases by one pathologist with the remaining 42 by the other pathologist. All 119 scans were examined by the anthropologist and the radiologist.

Once the data transfer system was established and the backlog of cases sent (15 cases were sent between 15 and 24 h after the scan on days 3 and 4), the average time from completing the scan to receiving confirmation the scan had been sent was 2 h 26 min (range 0:17–17:33). A small delay (approximately 10 min) was inherent in the process as the scans had to be saved to a compressed ‘zipped’ file and then the file taken to a point, separate from the scanner, for upload. Three scans were sent on the day after the scan was completed, as the site was closed on some days, even if work was not completed, due to concerns of noise pollution from local residents. Excluding these 3 scans, the mean time-to-send was 1 h 41 min (range 0:17–4:44). Staffing levels had the greatest effect on time-to-send as priority was given to scanning if only two radiographers were on-site. Data transfer also was not prioritised when the reporting team was not available until the following day.

In 78 cases (65%), the prototype ‘INTERPOL’ DVI form was completed by the pathologists on the same day as receipt of the scans with 35 cases completed the following day (96% of cases had the completed within 1 working day of image receipt). The remaining 5 cases were completed within 2 days of image receipt. Of these, 73 (61%) were sent electronically by the support staff team to London the same day as completion with 30 sent the following day. The remaining 15 were sent within 5 days of completion. Seventy-eight cases (65%) had the electronic reconstructed case images sent with the DVI form on the same day of image receipt. A further 9 electronic reconstructed case image datasets were sent without a DVI form on the same day of receipt with the remaining cases sent the next day. Thus, with a single case exception which was delayed by 8 days, all electronic reconstructed case image datasets were sent within 1 day of image receipt.

Seventy-five cases (63%) had preliminary typed radiology reports completed and sent by secure electronic transfer to London on the first day of image receipt with 26 cases typed and sent the following day. The remaining 17 cases were typed and sent to London between 2 and 15 days after image receipt. The preliminary reports were then checked by the radiologist. The final agreed reports were then sent to London. Three were sent on the same day of image receipt, 6 the following day, 28 between 2 and 7 days, 53 between 2 and 4 weeks and 28 up to 9 weeks later. Receipt of all reports and reconstructed case images was recorded after confirmation by the support team in Leicester.

### Reporting forms

For each case, two separate reporting forms were generated at the time of the image assessment.

The first was the completion of the prototype ‘INTERPOL’ radiology reporting form. Prior to the Grenfell Tower disaster, there were no means within the INTERPOL Pink forms of recording the findings of a radiology examination. A concept form had been in development, initiated by Professor Guy Rutty, EMFPU, Chair of the ISFRI DVI working group, assisted by Dr. Chris O’Donnell, forensic radiologist, Victorian Institute of Forensic Medicine, Melbourne, Australia, Chair ISFRI, and Mr. Mark Viner, forensic radiographer, International Association of Forensic Radiographers (IAFR). This prototype form was initially used and then modified by the EMFPU reporting team, based on their experiences of reporting of all cases. The final version of the form was presented to and adopted by INTERPOL, following slight further modification, in May 2018 [12].

The second form used depended upon the state of the remains. Where a body was complete, almost complete or had recognisable organ-containing compartments present, for example the head, neck and thorax or the thorax, abdomen and pelvis then the routine EMFPU forensic radiology reporting form was used to record the external and internal identification and pathological findings. Where single or comingled bone fragment remains, or single body parts were present, a new ‘remains form’ was designed and used to record the findings.

The forms used are published in association with reference [12]. All reports were initially handwritten. They were then transcribed by the support staff team immediately after production and checked by the report author as soon as they were typed. Preliminary electronic reports were sent to London to guide the investigation. The final report with any necessary amendments was then issued later after radiologist review.

### Report images

For each case, a set of reconstructed case images was produced to assist the DVI team in London. Images were produced by the reporting pathologist and anthropologist to demonstrate any reportable features. A separate index list of case images was produced. Our established ‘minimum image dataset’ for anthropological identification was developed for each case [13]. This included a 3D reconstruction of the external surface of the whole remains as well as a 3D reconstruction of the whole skeletal remains. Where possible a ‘virtual’ dental panoramic radiograph (OPT) was produced as well as individual images of teeth and reparative dental work. Depending on the case, image reconstructions were created to demonstrate potential identifiable factors including personal possessions, hazards and natural disease as well as epiphysis and internal sexual organs. The intention of these images was to provide a visual aid for the DVI and pathology teams.

Following the reporting of the cases, composite images of each flat were created by the forensic anthropologist. These images contained an overview of every body part/bone fragment that had been recovered from each flat. This process consolidated the image datasets and provided pathologists and anthropologists in the mortuary with a summary of what had been recovered, on a flat by flat basis.

## Discussion

We developed an efficient DVI scanning process that enabled all scans and paperwork to meet the coroner’s and police requirements. Conventionally, most non-specialist networks are focussed on high download speed, rather than upload speed. However, we were able to establish better network upload speed with the assistance of the BT Emergency Response Team. Wireless networks were tried, but due to the large amount of data being transferred and the intermittent connectivity, this proved impractical.

All scans were reported by a multiprofessional remote reporting team, which is adaptable to the needs of the disaster. For example, general experience in post-mortem imaging may be enhanced by introducing team members with sub-specialist expertise, including paediatrics, neuroradiology or significant traumatic injury. On this occasion, due to many cases with extreme heat damage, an anthropologist with PMCT experience proved invaluable.

The reporting team were able to provide a prototype INTERPOL DVI form along with a full report without the necessity to be present at the mortuary location. The intention of the reporting team was to anticipate the needs of the mortuary DVI teams by providing a prototype INTERPOL DVI form, a preliminary radiology report and a set of CT images prior to the examination of the remains. In attempting to achieve this, a prototype form was completed by the radiographers at the time of scanning. The remote reporting team then completed and sent 96% of prototype DVI forms, 99% of image datasets and 86% of preliminary reports within one working day of image receipt. Since this disaster, INTERPOL has adopted the radiology reporting form into the latest version of the Pink forms (https://www.interpol.int/en/How-we-work/Forensics/Disaster-Victim-Identification-DVI last visited April 2019). ISFRI has published a positional statement on this and other reporting forms for radiology reporting in DVI [12], and the Polish Society of Forensic Medicine and Criminology has published a positional statement recommending the use of the INTERPOL radiology reporting form [14].

Communication by phone between the mortuary and remote reporting teams was established to allow resolution of any queries to be addressed immediately. The reconstruction cases’ image datasets were provided to the DVI teams in the mortuary. In and post-event feedback informed the remote reporting team that the reconstructed images provided assisted the mortuary DVI teams.

Where the PMCT report is used to replace an invasive autopsy examination, it is required urgently to facilitate body release to relatives. As all the Grenfell Tower victims underwent autopsy and anthropological examination, a decision was made that the final full report was not required urgently, hence the longer interval before issuing the final radiology-checked reports. In other DVI incidents, rapid radiologist reporting turnaround has been achieved using on-site radiologists. However, for a disaster of the size of Grenfell Tower, a larger radiologist team than previously used would be required to achieve this goal, which may interfere with clinical diagnostic reporting. This consideration is particularly important in mass disasters, where clinical services may also be expected to be under pressure. To avoid this potential problem, recruitment of international PMCT-experienced reporting colleagues could be considered, as exercised in 2011 during Exercise Hounslow. ‘Remote international reporting’ could have additional benefit of exploiting time zone differences to allow overnight reporting, a practice in increasing clinical use to avoid mistakes from tired staff.

Having a PMCT reporting team in the mortuary may have been beneficial. This would have enabled those forensic specialists working in the mortuary who were not familiar with PMCT to benefit from the knowledge of the reporting team. However, with work commitments and the protracted time of this investigation, this would not have been feasible, particularly due to the lack of office space available on-site. The team makeup may also play a part of this decision as radiologist, for example, may not be used to working within a disaster mortuary environment. Not providing an on-site reporting service enabled the reporting team to continue with their urgent work commitments and so not compromising services in Leicester but at the same time provide the required radiological DVI information to the mortuary teams.

Key to the function of the remote team was the support staff of the EMFPU. Often forgotten in the planning for a mass fatality response, these team members supported the reporting team throughout, ranging from communication with the police, mortuary and coroner officer to report preparation, image packaging, secure data exchange, filing and data process logging—all critical processes within a robust medico-legal process.

Finally, even though the reporting team may be remote from the mortuary, consideration should be given to providing the team with appropriate welfare support. Working in a disaster DVI environment, even for those familiar with forensic work, may place an additional psychological burden upon them, and thus real-time and post-disaster welfare support should be factored into any work environment to try and ensure that individuals do not succumb to post-traumatic distress syndrome.

## Conclusions

PMCT has now been used in a variety of mass fatality incidents. Where an on-site radiology reporting team may not be available, we have demonstrated that an efficient DVI radiology reporting system can be established by use of a remote reporting team with supporting clerical staff without compromising the DVI or medico-legal investigations or local autopsy services. Key to this process is the early establishment of a secure, data transfer system. For those involved in planning for mass fatality incidents, we recommend that provision for DICOM data transfer for remote reporting should be built into local resilience plans.

## Electronic supplementary material

ESM 1 (PDF 223 kb)
