# Supplementary figures and images for: Remote post-mortem radiology reporting in disaster victim identification: experience gained in the 2017 Grenfell Tower disaster
Source: Int J Legal Med. 2019 Jun 28;134(2):637–43. doi: 10.1007/s00414-019-02109-x (PMC7044252; doi:10.1007/s00414-019-02109-x)

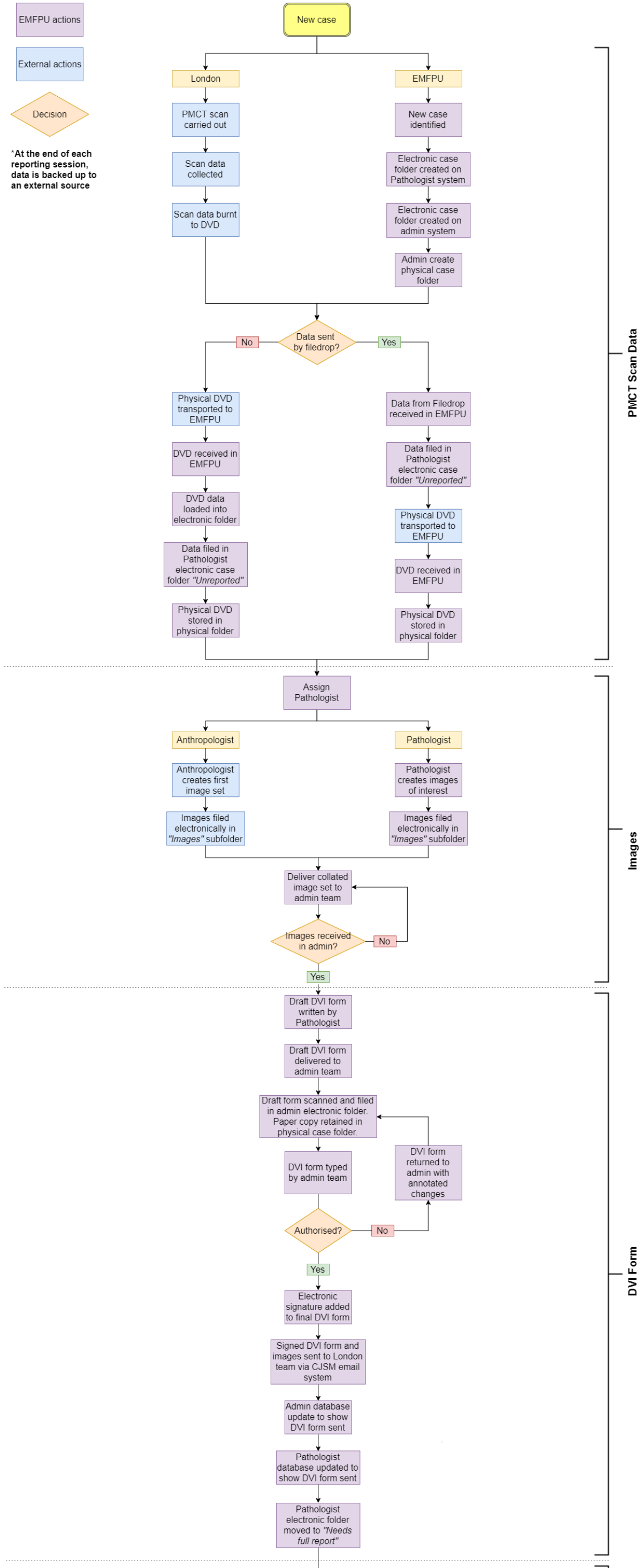

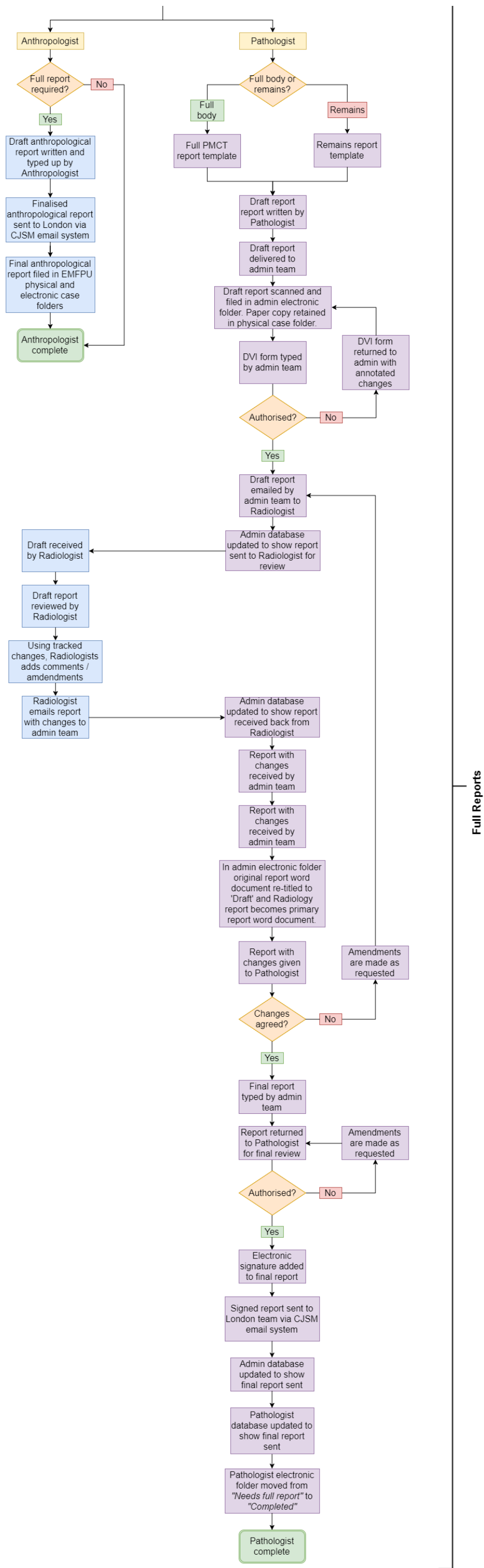

Supplement: Supplementary file 1 — (PDF 223 kb) [file 414_2019_2109_MOESM1_ESM.pdf]
